# Organochlorine pesticide level in patients with chronic kidney disease of unknown etiology and its association with renal function

**DOI:** 10.1186/s12199-017-0660-5

**Published:** 2017-05-26

**Authors:** Rishila Ghosh, Manushi Siddarth, Neeru Singh, Vipin Tyagi, Pawan Kumar Kare, Basu Dev Banerjee, Om Prakash Kalra, Ashok Kumar Tripathi

**Affiliations:** 1 Department of Biochemistry, Environmental Biochemistry and Immunology Laboratory, University College of Medical Sciences (University of Delhi) and G.T.B. Hospital, Dilshad Garden, Delhi, 110095 India; 2 Multidisciplinary Research Unit, University College of Medical Sciences (University of Delhi) and G.T.B. Hospital, Dilshad Garden, Delhi, 110095 India; 3 Department of Medicine, University College of Medical Sciences (University of Delhi) and G.T.B. Hospital, Dilshad Garden, Delhi, 110095 India

**Keywords:** Organochlorine pesticides, Chronic kidney disease of unknown etiology, eGFR, Urinary albumin

## Abstract

**Background:**

Involvement of agrochemicals have been suggested in the development of chronic kidney disease of unknown etiology (CKDu). The association between CKDu and blood level of organochlorine pesticides (OCPs) in CKDu patients has been examined in the present study.

**Methods:**

All the recruited study subjects (*n* = 300) were divided in three groups, namely, healthy control (*n* = 100), patients with chronic kidney disease of unknown etiology (*n* = 100), and patients with chronic kidney disease of known etiology (CKDk) (*n* = 100). Blood OCP levels of all three study groups were analyzed by gas chromatography.

**Results:**

Increased level of OCPs, namely α-HCH, aldrin, and β-endosulfan, were observed in CKDu patients as compared to healthy control and CKD patients of known etiology. The levels of these pesticides significantly correlated negatively with the estimated glomerular filtration rate (eGFR) and positively with urinary albumin of CKD patients. Logistic regression analysis revealed association of γ-HCH, *p*, *p*′-DDE, and β-endosulfan with CKDu on adjustment of age, sex, BMI, and total lipid content.

**Conclusions:**

Increased blood level of certain organochlorine pesticides is associated with the development of chronic kidney disease of unknown etiology.

## Background

Chronic kidney disease (CKD) is a complex pathophysiologic process with multiple etiologies frequently leading to end-stage renal disease (ESRD). Currently, 10% of the global population regardless of ethnic origin is affected by chronic kidney disease. CKD is characterized by progressive loss of functional glomerular tissue, defects in the glomerular filter function, and subsequent proteinuria [1]. It shares a common appearance of glomerulosclerosis, vascular sclerosis, and tubulointerstitial fibrosis, eventually causing scarring and nephron loss, thereby perpetuating a vicious cycle that results in the end-stage kidney disease [1].

CKD may develop as a consequence of (i) systemic disease such as diabetes (30.6%) and hypertension (13.2%), (ii) glomerulonephritis (9.9%), and (iii) other causes including the action of drugs, toxins and metals, infections, mechanical damage, ischemia, obstruction of the urinary tract, and genetic alterations [1]. However, in significant number of CKD cases, the underlying cause remained unknown. In the recent report of CKD registry of India, chronic kidney disease of undetermined etiology (CKDu) was found to be the second most common cause [2]. CKDu patients are characterized by non-conformation to the known risk factors such as diabetes, hypertension, or chronic glomerulonephritis. Although no significant literature is available which suggest the causes of CKDu, recently, some reports from El Salvador [3], Central America [4], Mexico [5], and Sri Lanka [6] have suggested the possible involvement of agrochemicals in the development of CKDu.

Organochlorine pesticides are a group of agrochemicals that were used extensively in yester year for pest control. Although majority of organochlorine pesticides (OCPs) are banned in most of the countries, yet they persist in the environment due to their long half-life and human exposure has been detected in general population in various geographical regions around the world.

Carreno et al. has detected 14 different OCPs among young males from Southern Spain and have reported all the blood samples contain *p*, *p*′-dichlorodiphenyldichloroethylene (*p*, *p*′-DDE) and endosulfan in them [7]. In another report, various OCPs mainly β-HCH, hexachlorobenzene (HCB), and *p*, *p*′-DDE were detected among the participants of Canadian study of health and aging [8]. In a recent publication, hexachlorocyclohexane (HCH), aldrin, α- and β-endosulfan, HCB, *p*, *p*′-dichlorodiphenyltrichloroethane (*p*, *p*′-DDT), and *p*, *p*′-DDE have been detected in mother’s plasma as well as in the umbilical cord plasma among mother-infant pairs from rural Mexico [9]. Recently, presence of HCH, *p*, *p*′-DDT, *p*, *p*′-DDE, heptachlor, β-endosulfan, and endrin aldehyde have been reported among the inhabitants of Southern Mexico and *p*, *p*′-DDE and β-endosulfan are the most frequently found OCPs in them [5].

Exposure to OCPs has been shown to be associated with adverse reproductive outcome [10], cardiovascular disease [11], and diabetes mellitus [12]. Some of the OCPs are nephrotoxic, and therefore, it is possible that body burden of OCPs may alter renal function leading to development of CKD of unknown etiology. Hence, in this study, we determined the level of organochlorine pesticides in CKDu and CKD patients of known etiology (CKDk) and also correlated the blood levels of OCPs with the glomerular filtration rate and urinary albumin of these patients in order to examine whether an association between OCP level and occurrence of CKDu exists.

## Methods

### Cases and controls

We recruited 100 patients each of CKDk and CKDu, aged between 30 and 50 years among the patients who attended to the nephrology clinic at University College of Medical Sciences and Guru Teg Bahadur Hospital (G.T.B.H.), Delhi, India, during the period of January 2014 to March 2015. CKD was defined as deranged renal function for more than 3 months with/without evidence of proteinuria and having estimated glomerular filtration rate (eGFR) ≤60 mL/min at least on two different occasions 3 months apart [13]. CKD patients of known etiology (CKDk) were having established etiology such as diabetes (54%), hypertension (32%), and glomerulonephritis (14%). Patients were diagnosed as having CKDu on the basis of WHO guidelines [14]. CKD is considered as of unknown origin in the absence of a past history of diabetes mellitus, chronic or severe hypertension, snake bite, glomerulonephritis or urological disease, normal HBA_1_C (<6.5%), and blood pressure <160/100 mmHg untreated or <140/90 mmHg on up to two anti-hypertensive medications. Age-sex-matched healthy controls (*n* = 100) were recruited mostly from nonrelated persons accompanying different patients at the renal clinic and staff of the hospital. Age matching was done in intervals of 5 years. Subjects occupationally exposed to pesticides and industrial chemicals such as those belonging to farming communities were also excluded. The study was approved by the Institutional Ethics Committee for Human Research (IEC-HR) of University College of Medical Sciences. Written informed consent was obtained from all participants. Routine investigations including different lipid fractions (triglycerides, total cholesterol, LDL, cholesterol, and HDL cholesterol) were measured at the Hospital Laboratory Services (HLS) at GTB Hospital, Delhi. Body mass index (BMI) was measured by weight/height^2^ (kg/m^2^). Total serum lipid levels were calculated by using the formula: total lipids = 2.27 total cholesterol + triglycerides + 0.623 [15].

### Collection and storage of blood samples

Venous blood samples were drawn after overnight fasting and collected in sterile EDTA containing vials. Immediately after sampling, 3 mL of blood was sent to HLS for routine biochemical analysis and 2 mL of whole blood was used for pesticide extraction.

### Estimation of organochlorine pesticides

Extraction of OCPs from blood was done in duplicate by using HPLC grade hexane and acetone (1:1) according to the method of Bush et al. followed by cleaning up procedure which was done by USEPA method using florisil [16]. In brief, 2 mL of blood was mixed with 10 mL of hexane and acetone (1:1) mixture and were shaken vigorously in a mechanical shaker for 30 min at room temperature. The extract was centrifuged for 10 min at 2000 rpm, and the clear hexane layer was separated. The extraction procedure was repeated twice, and the extracted hexane layer was pooled. Clean up was done by USEPA method 3620B using florisil (Sigma-Aldrich, USA) by column chromatography. The eluted hexane was concentrated by evaporation, and the concentrated residues were dissolved in 1 mL HPLC grade hexane for analysis.

### Gas chromatography analysis

Quantification of OCP residue levels was done by using Perkin Elmer Gas Chromatograph equipped with Ni^63^electron capture detector as described by Siddharth et al. [17]. Elite-GCDB-5 columns, 60 m long and 0.25 mm internal diameters, were used. One microliter of final extract was injected at a temperature of 170 °C with 1-min hold time. Thereafter, the temperature was raised from 170 to 225 °C at a rate of 5 °C/min with a 5-min hold time and finally raised from 225 to 275 °C at a rate of 6 °C /min with a 15-min hold time. The total run length was 40 min per sample. Quantitative analysis of all component residues in each sample was done by comparing the peak area with those obtained from a chromatogram of a mixed OCP standard of known concentration (Sigma-Aldrich Company, USA). Limits of detection for all OCPs were 4 pg/mL. Data below the detection unit were reported as 0.002 by default and was included for statistical analysis. Few blood samples in triplicate were spiked with a mixed standard of organochlorine pesticides (Sigma-Aldrich Company, USA), ranging between 5 and 25 ng/mL. The average recoveries of fortified samples exceeded 95%. Also, a quality check sample was always run with each set of samples for pesticide analysis to maintain accuracy.

### Statistical analysis

Statistical analysis was carried out using SPSS software version 17.0. Normally distributed data were expressed as mean ± SD, and the three groups were compared using one-way ANOVA followed by Tukey’s test. Non-parametric data such as urinary albumin excretion and blood level of OCPs were expressed by median and interquartile range. Kruskal-Wallis test was applied for comparison among three groups. Relationships between OCPs and eGFR were tested using Spearmen’s correlation analysis. For association of OCPs with risk of CKD, blood level of OCPs was distributed in tertiles using control group tertile as cutoff points and binary logistic regression analysis was used to calculate the risk of CKD. Odds ratio was adjusted for age, sex, BMI, and total lipid content. We analyzed the data in three different models (model 1: control vs CKDk, model 2: control vs CKDu, and model 3: CKDk vs CKDu).

## Results

The present study was carried out using three groups of study subjects, namely CKD patients of unknown etiology (CKDu), CKD patients with known etiology (CKD_k_), and healthy control subjects. CKD patients enrolled in this study mostly were in stage 3 or 4 as per National Kidney Foundation Kidney Disease Outcomes Quality Initiative (NKFKDOQI) classification. CKD patient of known etiology were mostly having nephropathy along with retinopathy due to diabetes mellitus.

### Demographic data of study subjects

Demographic data of the enrolled subjects are shown in Table 1. It can be observed that the study groups were more or less similar in terms of demographical characteristics such as age, sex, and BMI. The blood pressure, levels of total cholesterol, and triglyceride were found higher in CKDk/CKDu patients as compared to healthy controls; however, the increase was found to be statistically insignificant. Renal function parameters, namely serum creatinine, eGFR, blood urea, and urinary albumin were found significantly higher in enrolled CKD/CKDu patients as compared to healthy controls.

**Table 1:** Demographic features and biochemical parameters of the study subjects

Characteristics	Healthy controls (*n* = 100)	CKDk patients (*n* = 100)	CKDu patients (*n* = 100)	One-way ANOVA	Significance
	I	II	III		
Age (years)	42.76 ± 12.6	43.73 ± 11.3	45.23 ± 12.5	0.135	
Sex (M/F)	60:40	60:40	60:40		
BMI (kg/m^2^)	22.06 ± 1.8	21.94 ± 3.2	21.46 ± 2.1	0.621	
Blood pressure (mmHg) SBP	119.07 ± 9.1	137.8 ± 15.2	128.8 ± 11.2	0.00*	I vs II = 0.000
					I vs III = 0.000
					II vs III = 0.000
Blood pressure (mmHg) DBP	75.37 ± 4.4	90.6 ± 9.5	74.47 ± 7.3	0.00*	I vs II = 0.000
					I vs III = 0.113
					II vs III = 0.001
Fasting plasma glucose (mg/dL)	91.83 ± 5.63	148.83 ± 5.23	92.10 ± 4.36	0.044*	I vs II = 0.000
					I vs III = 0.999
					II vs III = 0.000
Post prandial plasma glucose (mg/dL)	119.2 ± 4.26	197.43 ± 6.38	115 ± 3.25	0.029*	I vs II = 0.000
					I vs III = 0.903
					II vs III = 0.000
Total cholesterol (mg/dL)	160 ± 8.1	189.74 ± 16.5	190.9 ± 18.2	0.004*	I vs II = 0.000
					I vs III = 0.684
					II vs III = 0.000
Triglyceride (mg/dL)	86.3 ± 6.9	148.28 ± 11.7	129.97 ± 16.8	0.001*	I vs II = 0.023
					I vs III = 0.01
					II vs III = 0.04
Total lipid (mg/dL)	519.82 ± 25.6	600.52 ± 10.9	612.2 ± 11.5	0.005*	I vs II = 0.001
					I vs III = 0.01
					II vs III = 0.04
Blood urea (mg/dL)	20.8 ± 4.7	65.5 ± 18.0	70.8 ± 18.8	0.000*	I vs II = 0.000
					I vs III = 0.000
					II vs III = 0.097
eGFR (mL/min/1.73 m^2^)	99.2 ± 10.8	57.5 ± 9.4	59.3 ± 10.5	0.000*	I vs II = 0.004
					I vs III = 0.001
					II vs III = 0.321
Urinary albumin excretion mg/dL (24 h), median (25–75th percentile)^a^	26.0 (22.0–30.0)	850.0 (377.5–1925.0)	870.0 (350.0–1630.0)	0.008*	I vs II = 0.000 I vs III = 0.000 II vs III = 0.729

All data represents the mean ± SD; one-way ANOVA with post hoc Tukey test was applied for significance test

*p value is significant at <0.05

^a^Kruskal-Wallis test was applied for comparison of 24 h urinary albumin excretion data

### Blood levels of organochlorine pesticides

Following pesticide extraction from blood samples and analysis by gas chromatography, we detected nine OCPs, namely the different isomeric forms of HCH (α, β, γ) and endosulfan (α, β), aldrin, dieldrin along with *p*, *p*′-DDT and its metabolite DDE in both group of CKD patients and in healthy controls. Considering the non-parametric nature of blood organochlorine pesticide levels in blood of the study subjects expressed in parts per billion (ppb), the results were depicted as median and interquartile range as shown in Table 2. In CKDk patients, the level of two pesticides, namely α-HCH and *p*, *p*′-DDE, were found significantly higher as compared to healthy control, whereas in CKDu patients, significantly increased level of four pesticides, namely α-HCH, aldrin, β-endosulfan, and *p*, *p*′-DDE, were found as compared to healthy controls. When the blood level of pesticides were compared between CKDk and CKDu patients, increased concentration of two pesticides, namely β-endosulfan and *p*, *p*′-DDE, were found in CKDu patients.

**Table 2:** Level of organochlorine pesticides in study subjects

OCPs (ppb)	Group I healthy controls (*n* = 100)	Group II CKDk patients (*n* = 100)	Group III CKDu patients (*n* = 100)	Kruskal-Wallis test	Significance
	Median (25th–75th percentile)	Median (25th–75th percentile)	Median (25th–75th percentile)		(Bonferroni adjusted *p* value)
α-HCH	0.7 (0.002–1.66)	1.26 (0.34–3.15)	1.68 (0.12–4.26)	0.004*	I vs II = 0.02
					I vs III = 0.01
					II vs III = 0.06
β-HCH	1.7 (0.002–3.96)	2.49 (0.84–4.65)	2.15 (0.64–5.16)	0.561	I vs II = 0.265
					I vs III = 0.341
					II vs III = 0.685
γ-HCH	2.6 (0.002–3.21)	2.15 (0.64–4.23)	2.03 (0.002–2.49)	0.236	I vs II = 0.369
					I vs III = 0.215
					II vs III = 0.347
Aldrin	1.6 (0.002–2.15)	1.96 (0.002–3.12)	2.15 (0.002–3.18)	0.045*	I vs II = 0.064
					I vs III = 0.045
					II vs III = 0.896
Dieldrin	2.5 (0.002–3.25)	0.89 (0.002–2.01)	1.95 (0.26–4.36)	0.412	I vs II = 0.452
					I vs III = 0.426
					II vs III = 0.439
α-endosulfan	0.7 (0.002–1.12)	0.49 (0.002–1.32)	2.18 (0.28–4.59)	0.321	I vs II = 0.365
					I vs III = 0.254
					II vs III = 0.615
β-endosulfan	1.3 (0.002–2.65)	0.84 (0.002–1.54)	2.38 (0.65–4.28)	0.001*	I vs II = 0.055
					I vs III = 0.042
					II vs III = 0.012
*p*, *p′*-DDT	1.2 (0.002–2.15)	0.23 (0.002–0.23)	2.36 (0.95–4.66)	0.061	I vs II = 0.321
					I vs III = 0.354
					II vs III = 0.316
*p*, *p′*-DDE	2.6 (0.002–3.54)	1.54 (0.29–2.64)	2.94 (0.68–4.58)	0.03*	I vs II = 0.01
					I vs III = 0.04
					II vs III = 0.03

Kruskal-Wallis test was applied to compare the OCP data in different groups

*Significance level *p* < 0.05

### Correlation of eGFR and urinary albumin excretion with blood OCPs level

Correlation analysis of eGFR and 24-h urinary albumin excretion with blood OCP level is presented in Table 3. In CKDu patients, four OCPs, namely γ-HCH, aldrin, β-endosulfan, and *p*, *p*′-DDE, exhibited significant negative correlation with eGFR, whereas in CKDk patients, only two OCPs, namely γ-HCH and *p*, *p*′-DDE, significantly and negatively correlated with eGFR. These pesticides also showed significant positive correlation with 24-h urinary albumin excretion (UAE) indicating that as the pesticide level increases eGFR tend to decrease with concomitant increase in UAE.

**Table 3:** Spearman’s correlation coefficient between OCP levels, eGFR, and urinary albumin excretion of CKD patients

OCPs (ppb)		Correlation coefficient (*ρ*) in CKDk patients	Correlation coefficient (*ρ*) in CKDu patients
α-HCH	eGFR	−0.11	−0.152
	UAE	0.103	0.132
β-HCH	eGFR	−0.145	−0.162
	UAE	0.163	0.156
γ-HCH	eGFR	−0.151*	−0.199*
	UAE	0.251*	0.269*
Aldrin	eGFR	−0.123	−0.194*
	UAE	0.143	0.204*
Dieldrin	eGFR	−0.111	−0.129
	UAE	0.159	0.164
α-endosulfan	eGFR	−0.132	−0.163
	UAE	0.152	0.145
β-endosulfan	eGFR	−0.174	−0.201*
	UAE	0.158	0.216*
*p*, *p′*-DDT	eGFR	−0.162	−0.123
	UAE	0.172	0.168
*p*, *p′*-DDE	eGFR	−0.222*	−0.284*
	UAE	0.259*	0.256*

*eGFR* estimated glomerular filtration, *UAE* 24-h urinary albumin excretion

*Significance level *p* < 0.05

### Logistic regression analysis for association of pesticides with CKD

Binary logistic regression analysis was applied to find out the association of blood level of OCPs with CKD using first tertile data as reference. The results were presented as odds ratio and 95% CI (Table 4). No significant association was observed during binary logistic regression analysis of second tertile data using first tertile data as reference in all the three models of analysis. Only third tertile pesticide data showed some significant association. Model 1, examined the association of blood OCP level with a risk of CKDk taking healthy control as reference group. Only *p*, *p*′-DDE (OR = 1.63) showed significant association with CKDk. In model 2, the association of blood OCP level with the risk of CKDu was examined taking healthy control as reference group. Three pesticides, namely γ-HCH (OR = 2.05), β-endosulfan (OR = 1.92), and *p*, *p′*-DDE (OR = 2.13), showed significant association with CKDu. Model 3 examined association of blood OCP level with the risk of CKDu taking CKDk as reference group. Two pesticides, namely β-endosulfan (OR = 2.16) and *p*, *p*′-DDE (OR = 3.20), exhibited significant association with CKDu.

**Table 4:** Association of organochlorine pesticides with study subjects

OCP (ppb)		1st tertile	2nd tertile	3rd tertile
			OR (95% CI)	OR (95% CI)
α-HCH	Model 1	Referent	0.82 (0.63–1.25)	0.99 (0.83–1.55)
	Model 2		0.68 (0.54–1.49)	1.72 (0.83–4.58)
	Model 3		0.65 (0.43–1.48)	1.95 (0.93–5.18)
β-HCH	Model 1	Referent	0.66 (0.48–1.12)	1.72 (0.48–2.04)
	Model 2		0.79 (0.62–1.67)	1.66 (0.72–2.53)
	Model 3		0.57 (0.45–1.69)	1.29 (0.50–2.16)
γ-HCH	Model 1	Referent	0.52 (0.37–1.14)	1.82 (0.83–3.58)
	Model 2		0.78 (0.62–4.04)	2.05 (1.84–4.04)*
	Model 3		0.63 (0.37–1.0)	0.93 (0.77–3.30)
Aldrin	Model 1	Referent	0.28 (0.16–1.24)	1.61 (0.77–3.32)
	Model 2		0.64 (0.45–1.64)	1.05 (0.95–3.11)
	Model 3		0.85 (0.89–1.27)	1.15 (0.89–3.87)
Dieldrin	Model 1	Referent	0.48 (0.37–1.56)	0.96 (0.57–1.56)
	Model 2		0.84 (0.52–1.96)	0.52 (0.32–1.87)
	Model 3		0.94 (0.75–1.68)	0.89 (0.58–2.53)
α-endosulfan	Model 1	Referent	0.39 (0.17–1.66)	1.05 (0.92–3.53)
	Model 2		0.92 (0.64–1.62)	0.87 (0.38–2.49)
	Model 3		0.88 (0.52–2.18)	1.30 (0.63–2.67)
β-endosulfan	Model 1	Referent	0.58 (0.49–1.85)	1.41 (0.68–2.87)
	Model 2		0.48 (0.22–1.64)	1.92 (1.15–3.00)*
	Model 3		0.92 (0.85–2.67)	2.16 (1.68–4.86)*
*p*, *p′*-DDT	Model 1	Referent	0.64 (0.32–1.94)	0.95 (0.32–2.93)
	Model 2		0.64 (0.28–1.60)	1.71 (0.68–3.90)
	Model 3		1.56 (0.96–2.68)	1.02 (0.59–2.50)
*p*, *p′*-DDE	Model 1	Referent	0.49 (0.22–1.46)	1.63 (1.10–2.93)*
	Model 2		0.59 (0.52–1.64)	2.13 (1.02–4.47)*
	Model 3		1.87 (0.99–2.49)	3.20 (1.48–6.94)*

Model 1, association of blood level of OCPs with CKDk with reference to healthy controls; model 2, association of blood level of OCPs with CKDu with reference to healthy controls; model 3, association of blood level of OCPs with CKDu with reference to CKDk. Odds ratio were adjusted for age, sex, BMI, and total lipid content

*OR* odds ratio

*Significance *p* < 0.05

## Discussion

In the present study, we detected nine organochlorine pesticides in the blood samples of our study subjects. There are significant differences in the level of four pesticides, namely α-HCH, β-endosulfan, *p*, *p*′-DDE, and aldrin, in the two groups of CKD patients as compared to healthy control (Table 2). The quantum of OCPs present in CKDu patients are higher as compared to that present in CKDk patients indicating increased pesticide accumulation in CKDu patients. No significant report is available to compare the increased level of pesticides in CKDu patients as observed by us. In an early report, Rutten et al. had reported increased levels of *p*, *p*′-DDE and HCH in uremic patients although there is no mention of the type of CKD patients used in their study [18]. In a recent report, increased level of a number of OCPs has been observed in CKD patients. The authors enrolled non-diabetic CKD patients although they are not exclusively CKDu patients [19]. Therefore, it appears that CKD patients tend to have higher blood level of pesticides as compared to normal healthy subjects. The finding of increased levels of OCPs in CKD patients suggest involvement of OCPs with deranged renal function and development of CKDu. This is supported by the fact that blood level of OCPs showed significant negative correlation with eGFR of CKDu patients (Table 3). Also, logistic regression analysis showed significant association of some of the pesticides with CKDu revealing that accumulated pesticides may be a contributory factor towards development of CKDu.

It is difficult to put forward any mechanism to link OCP accumulation and development of CKD as no definite etiology have been assigned towards development of CKDu. A number of animal studies have demonstrated the nephrotoxic nature of some of the OCPs [20, 21]. Also in a recent report, association between blood OCP level and oxidative stress have been reported in CKD patients [19]. OCPs are known inducer of oxidative stress as evidenced by several animals and in vitro studies [22, 23]. Oxidative stress is prevalent in CKD patients and is considered to be an important pathogenic mechanism. Also, renal disease is associated with a graded increase in oxidative stress and can accelerate renal injury progression [24].

Therefore, it is possible that accumulated pesticides may induce oxidative stress leading to development of CKD. The view of OCP accumulation and occurrence of CKDu is further evidenced by the fact that biopsy reports of CKDu patients reveal tubulointerstitial injury [25]. In a recent review on effect of environmental chemicals on renal function, it has been reported that some environmental chemicals indeed alter GFR and induce proteinuria. These authors suggested environmental chemical-mediated oxidative stress as the possible mechanism behind renal dysfunction and proposed that excessive oxidative stress alters the podocyte cytoskeleton leading to albuminuria, podocyte loss, tubular injury, and finally tubulointerstitial fibrosis [26]. Also, it has recently been pointed out that organochlorine pesticide-mediated oxidative stress induce MAP kinase pathway that eventually may cause renal dysfunction [27].

## Conclusions

In conclusion, the present study reveals association between increased blood levels of certain organochlorine pesticides and the occurrence of chronic kidney disease of unknown etiology for the first time to the best of our knowledge among urban population of Delhi who are not directly involved in agricultural activity or manufacture of pesticides and are probably exposed through environmental contamination. However, the data does not prove causality of the association. Despite strong body of evidences to suggest, defining the precise role of OCPs in the development of CKDu remains a challenge till date. Prolonged cumulative exposure in conjugation with other contributory factors possibly lead to renal dysfunction and CKDu. Further studies with improved study design involving larger population are required for better understanding of the etio-pathogenesis of CKDu and the role of OCPs in this context.

## Abbreviations

BMI: Body mass index
CKDk: Chronic kidney disease of known etiology
CKDu: Chronic kidney disease of unknown etiology
eGFR: Estimated glomerular filtration rate
ESRD: End-stage renal disease
G.T.B.H.: Guru Teg Bahadur Hospital
HCB: Hexachlorobenzene
HCH: Hexachlorocyclohexane
HLS: Hospital Laboratory Services
IEC-HR: Institutional Ethics Committee for Human Research
MAP kinase: Mitogen-activated protein kinase
NKFKDOQI: National Kidney Foundation Kidney Disease Outcomes Quality Initiative
OCPs: Organochlorine pesticides
OR: Odds ratio
*p*, *p*′-DDE: *p*, *p*′-Dichlorodiphenyldichloroethylene
*p*, *p*′-DDT: *p*, *p*′-Dichlorodiphenyltrichloroethane
UAE: Urinary albumin excretion
WHO: World Health Organization
